# Application of caries assessment spectrum and treatment instrument for staging and evaluating treatment needs of an adult population - A hospital based cross-sectional study

**DOI:** 10.4314/ahs.v21i4.48

**Published:** 2021-12

**Authors:** Agrawal Vineet, Shah Nimisha

**Affiliations:** Department of Conservative Dentistry and Endodontics, Sumandeep Vidyapeeth Deemed to be University, Piparia, Vadodara, Gujarat State, India

**Keywords:** Adults, Caries Assessment Spectrum and Treatment, Dental Caries, Treatment needs

## Abstract

**Background:**

Application of caries assessment spectrum and treatment (CAST) instrument for staging and evaluating treatment needs of an adult population suffering from dental carious lesion

**Objectives:**

Study was conducted with aim of applying CAST instrument for staging adult urban Indian population according to severity of carious lesion and treatment needs required.

**Methods:**

Cross-sectional descriptive study was conducted on 300 adult patients. After training session and calibration of examiner, all patients were examined for the caries experience and CAST codes were recorded on a structured proforma.

**Results:**

Of 300 adult patients, 18% (54) adults have healthy dentition denoted by codes 0,1, and 2. 12 % and 15.3% adults were in reversible premorbidity stage (code 3) and morbidity stage (codes 4 and 5) respectively. The largest number of adults 29.7% were seen in serious morbidity stage (codes 6 and 7) followed by 21.3% adults in mortality stage (code 8) requiring either root canal treatment or extraction and replacement of teeth as treatment needs.

**Conclusion:**

CAST instrument has been found useful in staging adult population suffering with dental carious lesion and evaluating their treatment needs. Moreover, CAST is easy to apply for staging the carious lesion due its hierarchical structure and reporting results in an understandable manner.

## Introduction

Dental caries is caused by imbalance between demineralization and remineralization process around the tooth surface. The Global Burden of Disease Study 2017^1^ estimated that oral diseases affect close to 3.5 billion people worldwide, with caries of permanent teeth being the most common condition. Globally, it is estimated that 2.3 billion people suffer from caries of permanent teeth.1 Although there is a decline in incidence of dental caries in the developed countries, developing countries like India have seen an increase due to minimal access to oral healthcare facilities.^2^

Research has shown that dental caries is preventable, controllable and treatable disease. Reliable picture of a population suffering from a dental caries is prerequisite so that government and health institutions can develop an intervention and dental caries treatment programs in a planned manner. It is only possible if we have a complete caries assessment and detection instrument which covers total dental caries spectrum. Undoubtedly, most widely used instrument for caries detection in epidemiological studies is that of World Health Organization (WHO), Decayed, Missing, and Filled Teeth (DMFT).^3^ Despite DMFT being advantageous in terms of simplicity, there are major disadvantages such as lack of early caries detection in enamel, making it out of touch for use in current concepts of caries prevention and treatment.^4^

International Caries Detection and Assessment System (ICDAS-I & II)^5^,^6^ and Pulpal, Ulcerative, Fistula and Abscess index (PUFA)^7^ were developed to record carious lesions more accurately but they too have their share of problems. Pulpal involvement as a consequence of severe dental caries was not recorded by ICDAS whereas on other hand enaml, dentinal or root caries lesions was not recorded by PUFA as it was concerned with only extreme ends of the caries disease spectrum.^8^ Also, ICDAS being a two digit system was cumbersome in reporting results in epidemiological surveys.^9^ The caries assessment spectrum and treatment (CAST) proposed,^8^ describes in a hierarchical way, the complete range or spectrum of carious conditions, from the absence of carious lesions, to the presence of caries protection (sealant) and caries treatment (restoration), lesions in enamel and dentine, lesions penetrating the pulp and tissue surrounding the tooth (abscess/fistulae), and loss of teeth (Table 1). The hierarchical approach implies that a high CAST score is considered to represent more severe condition than a low CAST score. Assessment is performed visually and the use of compressed air is not required. The CAST has undergone face, content, construct validation and reproducibility tests and results are positive.^10^,^11^,^12^

**Table 1:** CAST codes and description

Characteristic	Code	Description
	0	Sound – no visible evidence of a distinct carious lesion is present
**Sealed**	1	Sealed – pits and fissures have been at least partially sealed with a sealant material
**Restored**	2	A cavity has been restored with an (in) direct restorative material currently without a dentine carious lesion and no fistula/ abscess present
**Enamel**	3	Distinct visual change in enamel – a clear carious related discoloration (white or brown color) is visible, including localized enamel breakdown without clinical visual signs of dentine involvement
**Dentine**	4	Internal caries related discoloration in dentine – the lesion appears as shadows of discolored dentine visible through enamel which may or may not exhibit a visible localized breakdown
	5	Distinct cavitation into dentine – no (expected) pulpal involvement is present
**Pulp**	6	Involvement of pulp chamber – distinct cavitation reaching the pulp chamber or only root fragments are present
	7	Abscess/fistula – a pus containing swelling or a pus releasing sinus tract related to a tooth with pulpal involvement due to dental caries is present
**Lost**	8	The tooth has been removed because of dental caries
**Others**	9	Does not match with any of the other categories

Reporting the data available from the CAST score codes, not only helps to find the prevalence of dental caries among population studied but also helps to differentiate population in different stages of severity of disease due to hierarchical order of CAST codes.^13^ Also, type of treatment needed whether preventive or intervention can be decided for population under study according to CAST codes.

Most of the studies^14^,^15^ till date conducted using CAST were done for primary teeth or mixed dentition on children's rather than in adult population and complete set of permanent teeth. Also, these studies have only checked prevalence of dental caries and there is no study in literature reporting staging and treatment need of dental caries in adult population using CAST. Therefore, current study was conducted with the aim of application of caries assessment spectrum and treatment (CAST) instrument for staging and evaluating treatment needs of an urban indian adults presenting at an outpatient hospital clinic.

## Methods

### Study setting

The study was conducted on dental patients visiting the outpatient clinic of conservative dentistry and endodontics department of Manubhai Patel Dental college and Hospital, Vadodara, India. This dental hospital is a tertiary health institution that serves both urban and rural communities. It is a charitable dental hospital with 6.21 acres campus situated in the heart of city Vadodara and has an average of 150 dental patients visiting daily for oral cavity and tooth related problems.

### Study design and population

Cross-sectional descriptive study was conducted on 300 adult male and female patients (age range 15 – 70 years) with dental problems visiting outpatient clinic (from October 2019 to March 2020) of conservative dentistry and endodontics department of Manubhai Patel Dental college and Hospital. Patients having all the permanent teeth till second molars fully erupted and no deciduous teeth present and who are ready to give the written consent were included in study. Patients who are mentally retarded, immuno-compromised, pregnant females, differently abled, undergoing radiation and orthodontic treatment were excluded from the study since these conditions may predispose patients to dental caries and also these patients may not cooperate during screening and examination.

### Ethics

The study was reviewed and approved by the institutional ethical committee (Ethical committee approval no: SVIEC/IN/DENT/PhD/18018). Informed consent was obtained from all the patients who were enrolled in the study.

### Sample size determination and sampling technique

Sample size calculation was done considering total average number of adult patients with dental caries being treated in department in a month at 95% confidence interval and 80% power. 300 adult male and female patients were randomly selected based on purposive sampling technique. Sampling frame can not be taken as all patients were not admitted or visiting the outpatient clinic at same time.

### Data Collection

Before collection of data, two examiners were trained and calibrated having experience of more than 7 years. This training session comprised theoretical and practical components. The study of literature provided by authors of CAST instrument comprised theoretical part, whereas practical part includes dental examination of 20 extracted teeth and scoring them according to CAST codes. Individual scores of both examiners were compared and where there is a difference, examiners discussed the scores until consensus was reached. For calibration, 20 patients were examined by both examiners twice at interval of 1 week using the CAST codes as shown in Table 1. Cronbach's alpha value for inter-examiner variability for pre-data was 0.970 and post-data after 1 week was 0.998, which indicates high level of internal consistency. Also, for intra-examiner variability, paired t test was performed which showed the p>0.05 for both the examiners, indicating excellent intraexaminer reproducibility.

A structured and validated proforma was prepared to collect and record the data. The armamentarium used for collection of data were sterile mouth mirror, CPI probe (a periodontal probe ending with a 0.5 mm ball) which will remove dental plaque or debris if present, tweezer and cotton rolls. The data was collected and caries condition was measured based on the CAST scores under adequate illumination. If two carious conditions were present on the same tooth, that is, superficial carious lesion in one pit and a deep one in another, the higher score was recorded. Neither radiographic examination nor drying of the teeth was carried out as per CAST instrument.

### Data processing and analysis

Data were checked and entered in to Excel sheet, then exported to Statistical Package for Social Sciences (SPSS) version 20 (IBM computers, USA) for analysis. The staging of dental caries was estimated by using simple descriptive summary statistics such as frequency and proportion. Tables and graphs were used to present the result of the analyzed data.

## Results

**Figure 1:**
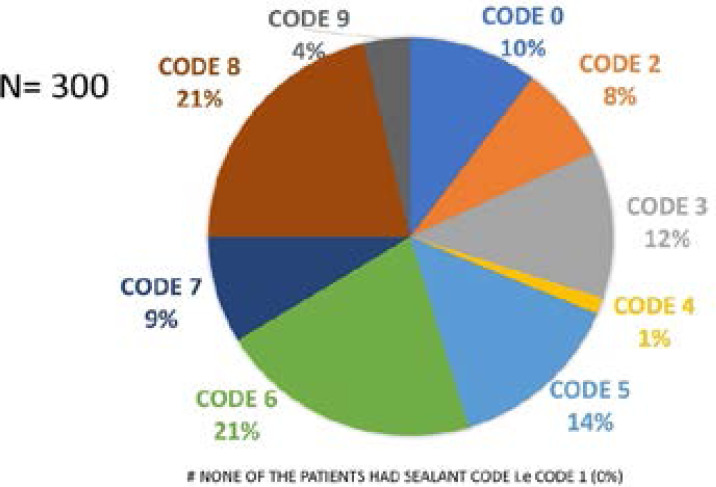
Frequency Distribution of Maximum CAST code per patient

Analysis of the codes of CAST index were done as suggested by Frencken et al. in 2013 and CAST manual. 16,17 The maximum CAST code per patient was derived and tabulated for frequency distribution as shown in Figure 1.

According to the developers of CAST index and on the basis of epidemiological concept of health and disease, findings can be descried as healthy dentition denoted by codes 0,1, and 2, this was observed in our study in 18% (31 and 23) adults. Nonhealthy dentition can be divided into:

1. A reversible premorbidity stage (code 3) seen in 12% (36) of the adults
2. Morbidity stage (codes 4 and 5) seen in 15.3% (4 and 42) of the adults
3. Serious morbidity stage (codes 6 and 7) – seen in 29.7% (63 and 26) of the adults and lastly
4. Mortality stage (code 8) most severe condition seen in 21.3% (64) of the adults.

**Figure 2:**
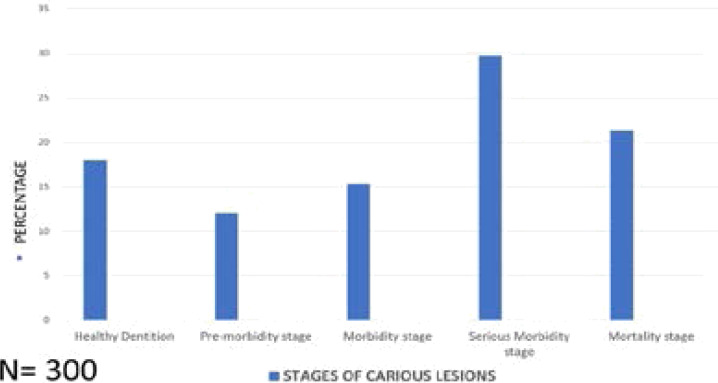
Staging of the patients depending on the extent of carious lesion

Graphical representation of all the stages can be observed in Figure 2.

Treatment needs of the patient can be classified based on the stages of the carious lesion as described in Table 2. Patients with healthy dentition (18%) does not require any treatment (Tn 0). Patients in pre-morbidity stage (12%) requires only preventive treatment (Tn1). Patients in morbidity stage (15.3%) requires intervention restorative treatment either intra or extra- coronal (Tn2). Patients in the serious morbidity (29.7%) require interventional root canal therapy (Tn3). Patients in mortality stage (21.3%) will require the replacement of the lost tooth either by prosthodontic crown and bridge procedure or by placing the dental implants (Tn4). Majority of the adult patients in our study are seen reporting in serious morbidity and mortality stages which requires either the root canal treatment or extraction (Tn3) and the replacement of the lost tooth (Tn4) (Table 2).

**Table 2:** Treatment needs according to carious stage

Characteristics	Codes	Stages	Treatment needs
	0	Healthy dentition	Tn0 – No treatment needed
**Sealed**	1		
**Restored**	2		
**Enamel**	3	Pre-morbidity stage	Tn1 – Restoration / Sealant application
**Dentine**	4	Morbidity stage	Tn2 – Restoration and indirect/direct pulp capping procedures
	5		
**Pulp**	6	Severe morbidity stage	Tn3 – Endodontic therapy / Extraction of teeth
	7		
**Lost**	8	Mortality stage	Tn4 – Replacement of teeth Crown and bridge or Dental implant

## Discussion

CAST is a novel instrument for epidemiological research studies comprising the full spectrum of caries-related conditions. CAST instrument is considered better than DMFT for epidemiological studies because it provides more detailed information on caries prevalence, stages of disease and the treatment needs. Also, compared to ICDAS, its use is less costly and time consuming. 4,18 Advanced process of its validation for face and content conducted via the RAND-modified e-Delphi method is added advantage of the CAST instrument.^10^

The hierarchical structure of the CAST instrument with the higher score assigned to more severe conditions makes it possible to stage the population according to the scores in different categorize and assign different treatment needs in the population to particular stage of caries. CAST instrument follows the modern epidemiological concept of health and disease and considers a restored tooth to be sound and well-functioning and highest possible score is given to teeth extracted due to caries as a symptom of caries mortality. 14,16

The present study is one of the first reports on staging and evaluating treatment needs of an adult population suffering from dental carious lesion using caries assessment spectrum and treatment instrument. At the time of preparation of this manuscript, no data on staging carious lesions in a population irrespective of age using CAST instrument has been found in the literature.

This staging allowed us to observe that maximum adult patients visiting the department was diagnosed to have at least one tooth with pulpal involvement i.e in the serious morbidity stage (29.7%) followed by patients who have lost at least one tooth due to dental caries i.e in mortality stage (21.3%). This result might be because all the patients were from the outpatient clinic of conservative dentistry and endodontics department of hospital and is not representative of a patient visiting general dentist.

Translating the above staging into the treatment needs, patients with healthy dentition does not require any treatment (Tn 0) but just the oral hygiene maintenance continuation such as regular tooth brushing and flossing. Patients in pre-morbidity stage requires the guidance regarding the oral health maintenance, diet counselling and also the restorative treatment and/or sealant application as prophylaxis (Tn1). Patients in morbidity stage requires a restorative treatment either intra or extra-coronal including the direct or indirect pulp capping procedures based on depth of the dentinal caries progression (Tn2). Patients in serious morbidity require root canal therapy (Tn3) provided that remaining tooth structure is restorable. If tooth structure is non-restorable such as only root fragments are remaining, extraction of teeth will be the treatment option. Patients in mortality stage will require replacement of lost tooth either by prosthodontic crown and bridge procedure or by placing dental implants (Tn4).^17^

The results of our study are consistent with the study conducted by Mehta A,^19^ who also showed similar prevalence of dental carious lesion in adult Indian population but author did not staged the population according to carious severity and treatment needs. In the absence of reports on CAST used to stage and evaluate treatment needs of a population, the present results of our studies can merely be compared with other studies. The data reported from our study will be helpful in preparing the department protocols, material stocks, patient appointment and treatment plans according to the number of patients in a particular stage of diseases. Future studies are recommended to be done on a larger scale using CAST instrument, so that policy makers, health planners and health professionals at national, regional, and district levels have an appropriate data regarding the stages and treatment needs of the entire population and can make policies and plans accordingly. There is a need to bring about a great awareness regarding oral hygiene maintenance, diet counselling and plaque control measures in our population so that they can be converted to healthy dentition stage gradually from advanced stages.

## Limitation

Sample is not representative of adult population in study area as it was obtained conveniently from Outpatient clinic of a dental department. Also, since only staging of the adult Indian population was aim of our study, the data collected would be helpful only at departmental or institutional level to categorize patients for treatment. For designing oral health programs and tracing their impact over time, prevalence studies are also required. Therefore, future studies are required in adult population on a larger scale and more representative sample.

## Conclusion

CAST instrument has been found useful in staging the adult population suffering with dental carious lesion and evaluating the treatment needs of such population. Moreover, CAST is easy to apply for staging the carious lesion due its hierarchical structure and reporting results is conducted in an understandable manner. Evaluating treatment needs of a population also became easy depending on the staging of carious lesion. More research into the use of CAST instrument for staging and treatment needs of different population suffering from dental caries are required on a larger scale.
